# Identification of Novel Biomarkers in Seasonal Allergic Rhinitis by Combining Proteomic, Multivariate and Pathway Analysis

**DOI:** 10.1371/journal.pone.0023563

**Published:** 2011-08-24

**Authors:** Hui Wang, Johan Gottfries, Fredrik Barrenäs, Mikael Benson

**Affiliations:** 1 The Unit for Clinical Systems Biology, University of Gothenburg, Gothenburg, Sweden; 2 Department of Chemistry, University of Gothenburg, Gothenburg, Sweden; 3 The Center for Individualized Medication, Linköping University, Linköping, Sweden; 4 Department of Pediatrics, Linköping University Hospital, Linköping, Sweden; National Institutes of Health, United States of America

## Abstract

**Background:**

Glucocorticoids (GCs) play a key role in the treatment of seasonal allergic rhinitis (SAR). However, some patients show a low response to GC treatment. We hypothesized that proteins that correlated to discrimination between symptomatic high and low responders (HR and LR) to GC treatment might be regulated by GCs and therefore suitable as biomarkers for GC treatment.

**Methodology/Principal Findings:**

We identified 953 nasal fluid proteins in symptomatic HR and LR with a LC MS/MS based-quantitative proteomics analysis and performed multivariate analysis to identify a combination of proteins that best separated symptomatic HR and LR. Pathway analysis showed that those proteins were most enriched in the *acute phase response pathway*. We prioritized candidate biomarkers for GC treatment based on the multivariate and pathway analysis. Next, we tested if those candidate biomarkers differed before and after GC treatment in nasal fluids from 40 patients with SAR using ELISA. Several proteins including ORM (P<0.0001), APOH (P<0.0001), FGA (P<0.01), CTSD (P<0.05) and SERPINB3 (P<0.05) differed significantly before and after GC treatment. Particularly, ORM (P<0.01), FGA (P<0.05) and APOH (P<0.01) that belonged to the *acute phase response pathway* decreased significantly in HR but not LR before and after GC treatment.

**Conclusions/Significance:**

We identified several novel biomarkers for GC treatment response in SAR with combined proteomics, multivariate and pathway analysis. The analytical principles may be generally applicable to identify biomarkers in clinical studies of complex diseases.

## Introduction

The beneficial effects of glucocorticoids (GCs) in the treatment of seasonal allergic rhinitis (SAR) are well established [1]. Despite this, 10–30% of patients with SAR and other allergic or autoimmune diseases show low or limited responses to GCs [2]. Hence, there is a clinical need to find biomarkers to monitor treatment response. The identification of such biomarkers is complicated by the large number of proteins that are involved in inflammatory diseases. Proteomics may help to detect and quantify those proteins, but the prioritization of candidate biomarkers is a challenge. Multivariate analysis can be used to prioritize combinations of biomarkers that best separate groups of patients. Pathway analysis helps to obtain a functional overview of those combinations and thereby further contributes to the prioritization [3]. Thus, employing a combination of proteomics, multivariate and pathway analysis to identify biomarkers for treatment response might be the solution, but the application of this combination in clinical studies is difficult. Many diseases have complex phenotypes, or it may not be possible to obtain samples from the affected organ. Another problem is that the evaluation of the effects of medication can be confounded by a variable disease course. SAR has several advantages as a disease model; symptoms occur at a defined time point during the year and are due to a known and measurable external factor, namely pollen. SAR is common, has a well-defined clinical phenotype and local inflammatory fluid is readily accessible. Several studies identified potential markers for treatment response in nasal fluids such as eosinophil cationic protein (ECP), alpha-2-macroglobulin (A2M) and albumin (ALB) [4], [5], [6], [7], [8]. However, considerable variations are observed. Other inflammatory diseases, including asthma demonstrate similar differences [2]. This may explain the variable response to GC treatment.

The aim of this study was to identify novel biomarkers for GC treatment response in SAR. We reasoned that patients who showed a high and low response to GC treatment (HR and LR, respectively) might be distinguished by differences in nasal fluid protein profiles, that might be targeted by GC treatment and therefore be potential biomarkers for GC treatment response. To find such proteins, we first profiled nasal fluids from symptomatic HR and LR with a quantitative proteomic analysis [9]. Next, we searched for the combination of proteins that best separated HR and LR, using multivariate analysis, namely orthogonal partial least squares-discriminant analysis (OPLS-DA) [10], [11]. We prioritized candidate biomarkers based on OPLS-DA modelling as well as pathway analysis [12], [13]. Finally, we validated these candidate biomarkers with ELISA in nasal fluids from patients with SAR before and after treatment. This led to identification of several novel biomarkers for GC treatment response.

## Results

### Characteristics of high and low responders to treatment with glucocorticoids

Forty symptomatic patients with SAR were recruited during the pollen season, and seen before and after nasal treatment with glucocorticoids (GCs). High responders (HR) were defined as the 10 patients with the greatest reduction in symptom scores following treatment with GCs, while low-responders (LR) were defined as the 10 patients with the lowest reduction (Table S1). The median (range) age of the 10 HR was 19 (18–47) and 6 were women. The mean ± SEM symptom score of HR after GC treatment decreased from 20.8±1.5 to 5.4±0.7 (P<0.01). The median (range) age of the LR was 19 (18–47) and 7 were women. The mean ± SEM symptom score of the LR increased from 14.5±1.9 to 18.4±1.8 (P<0.05). The mean ± SEM symptom score of HR and LR before GC treatment was 20.8±1.5 vs 14.5±1.9 (P<0.05).

### Quantitative proteomic analysis of nasal fluids from HR and LR

**Figure 1:**
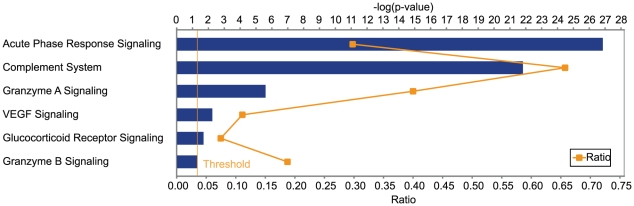
Pathways enriched with nasal fluid proteins identified in both HR and LR to treatment with Glucocorticoids (GCs). A total of 953 unique proteins were identified in nasal fluids from both HR and LR and were mapped onto canonical pathways using the IPA software. The yellow threshold indicates 95% confidence.

In order to profile proteins in nasal fluids from symptomatic HR and LR before treatment with GCs we performed a liquid chromatography–tandem mass spectrometry (LC-MS/MS) analysis with nasal fluids on a LTQ Orbitrap Velos instrument. We excluded two samples with extreme average signals, including one HR and one LR. With 99% confidence and one peptide as threshold, this led to the identification of 953 unique proteins. In order to obtain a functional overview of these identified proteins in both HR and LR, we performed pathway analysis with the Ingenuity Pathway Analysis (IPA) software. We found that the *acute phase response pathway* (P = 1.39×10^−27^, 54 proteins) and the *complement signalling* pathway (P = 1.52×10^−22^, 23 proteins) were most significantly enriched for nasal fluid proteins (Figure 1). Other inflammation-related pathways such as the *Granzyme A signalling pathway* and *Glucocorticoid receptor signalling* pathway were also significantly enriched (Figure 1).

### Multivariate analysis of differences in nasal fluid profiles between HR and LR

**Figure 2:**
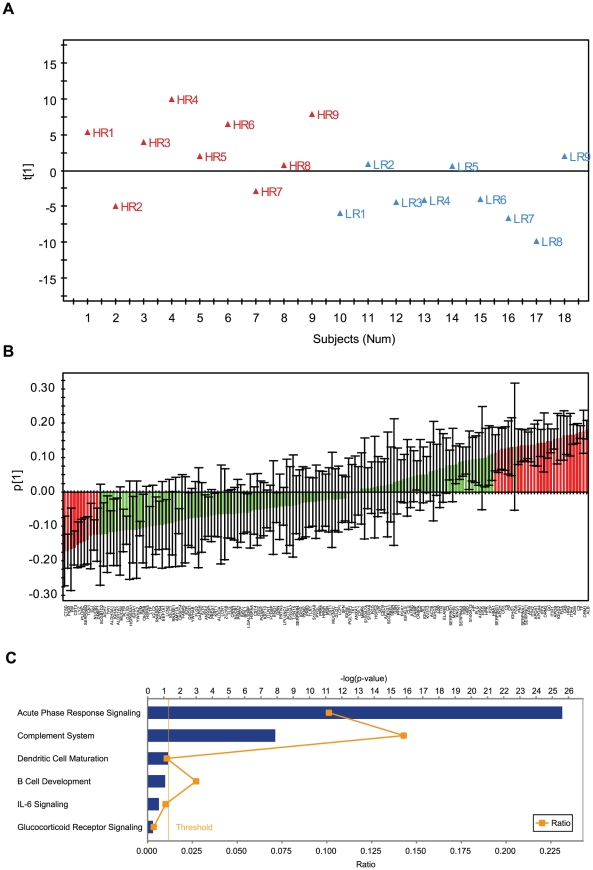
Differences in nasal fluid protein profiles between symptomatic HR and LR. OPLS-DA modelling was performed with 161 nasal fluid proteins identified in no less than 50% HR and LR. **A**) OPLS-DA score plot showed partial separation between HR and LR, where t[1] represents the predictive component. All samples were within a ±2 standard deviation (SD) limit (according to Hotelling's T^2^). **B**) OPLS-DA loading plot with confidence intervals (according to the cross validation procedure). The top 40 proteins that contributed to separation between HR and LR were highlighted in red. The black line represents error bar. **C**) Pathway analysis with the top 40 proteins using the IPA software. The yellow threshold indicates 95% confidence.

We searched for differences in nasal fluid protein profiles between symptomatic HR and LR using OPLS-DA modelling. Such an approach identifies correlation patterns that discriminate groups and estimates the relative importance of each included protein value for the discrimination [10], [11], [14]. We excluded proteins comprising more than 50% missing data in either HR or LR, which resulted in 161 proteins for modelling. OPLS-DA modelling with the 161 proteins provided a one predictive component model. The predictive variation between protein data and the discriminator response variable used 29% of the protein data (according to R^2^X) with an explained discrimination value of 38% (according to R^2^Y). The model was indicated to be significant according to the SIMCA software's default significance test (Cross-validation and Eigen vector size), and thus deemed to be useful for candidate biomarker selection, although with relatively low cross-validation (Q2<0.02). This indicated that HR and LR were partially discriminated (Figure 2A). Next, we extracted the top 40 proteins (25% of the input proteins in OPLS-DA modelling) that correlated to the discrimination between HR and LR, using OPLS-DA predictive loadings plot with significant confidence intervals (Figure 2B, Table S2).

To functionally overview the top 40 proteins that correlated to the discrimination between HR and LR, we performed pathway analysis using IPA. Pathway analysis showed that the *acute phase response pathway* (P = 2.45×10^−26^, 19 proteins) was significantly enriched (Figure 2C, Table 1). Of note, all the 19 proteins enriched in the *acute phase response pathway* increased in HR compared to LR (Table 1).

**Table 1:** Nasal fluid proteins enriched in the *acute phase response pathway*.

Protein ID	Protein symbol	Protein name	FC*
P01023	A2M	alpha-2-macroglobulin	1.36
P02768	ALB	Albumin	1.36
P02647	APOA1	apolipoprotein A-I	1.20
P02749	APOH	apolipoprotein H (beta-2-glycoprotein I)	1.76
P01024	C3	complement component 3	1.26
P0C0L5	C4	complement component 4B (Chido blood group)	1.29
P01031	C5	complement component 5	1.33
P00450	CP	ceruloplasmin (ferroxidase)	1.04
P02671	FGA	fibrinogen alpha chain	1.55
P02751	FN1	fibronectin 1	1.32
P02790	HPX	Hemopexin	1.14
P04196	HRG	histidine-rich glycoprotein	1.74
P19823	ITIH2	inter-alpha (globulin) inhibitor H2	1.61
Q14624	ITIH4	inter-alpha (globulin) inhibitor H4 (plasma Kallikrein-sensitive glycoprotein)	1.33
P19652	ORM2	orosomucoid 2	2.80
P02763	ORM1	orosomucoid 1	1.57
P01009	SERPINA1	serpin peptidase inhibitor, clade A (alpha-1 antiproteinase, antitrypsin), member 1	1.23
P05155	SERPING1	serpin peptidase inhibitor, clade G (C1 inhibitor), member 1	1.70
P02787	TF	Transferrin	1.31

*FC, fold change between HR and LR.

### Identification of biomarkers for GC treatment

We hypothesized that nasal fluid proteins that correlated to discrimination between HR and LR might be targeted by GC treatment and could be candidate biomarkers for GC treatment. We selected orosomucoid 1 (ORM 1), orosomucoid 2 (ORM2), apoliprotein H (ApoH), histidine-rich glycoprotein (HRG), albumin (ALB) and fibrinogen alpha chain (FGA) from the *acute phase response pathway* based on their estimated contribution to the discrimination between HR and LR in the OPLS-DA model (Figure 2C, Table S2). In addition, cathepsin D (CTSD), serpin peptidase inhibitor, clade B, member 3 (SERPINB3) and secretoglobin, family 1D, member 2 (SCGB1D2), which did not belong to the *acute phase response pathway*, were also selected as candidates based on their estimated contribution to the discrimination between HR and LR in the OPLS-DA model.

**Figure 3:**
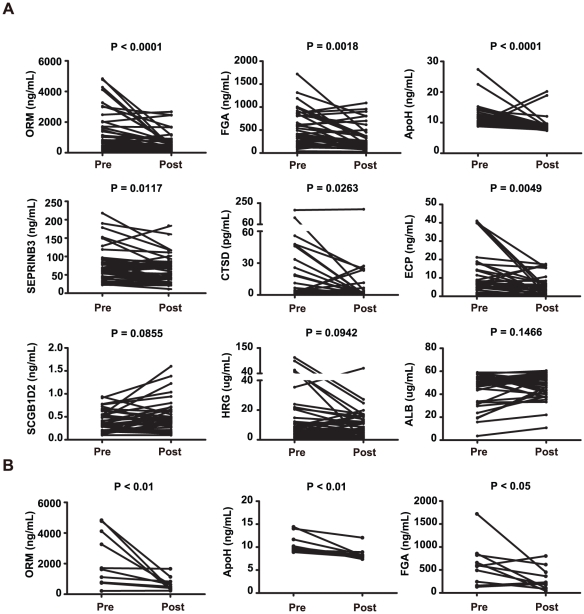
Identification of candidate biomarkers with ELISA. **A**) Nasal fluids from 40 patients with SAR before and after GC treatment were analyzed. **B**) Nasal fluids from 10 HR. Pre, patients before treatment with GCs; Post, patients after treatment with GCs.

We analyzed these proteins in nasal fluids from all 40 patients with SAR before and after GC treatment with ELISA. As a control, we first measured ECP, which is known to decrease in patients with SAR following treatment with GCs [12]. ECP decreased from 9.76±1.86 ng/mL before treatment to 4.75±0.82 ng/mL after treatment (P<0.01) (Figure 3A). Next, we measured the proteins identified by the combined modelling and pathway analysis. ORM (ORM1/ORM2) decreased from 1428.67±218.69 ng/mL before treatment to 656.84±104.67 ng/mL (P<0.0001). FGA decreased from 505.41±61.55 ng/mL before treatment to 319.42±43.00 ng/mL (P = 0.0018). APOH decreased from 11.76±0.57 ng/mL before treatment to 9.13±0.41 ng/mL (P<0.0001). SERPINB3 decreased from 77.39±7.35 ng/mL before treatment to 66.03±5.82 ng/mL (P = 0.0117). CTSD decreased from 17.01±5.70 pg/mL before treatment to 8.82±4.91 pg/mL (P = 0.0263). However, the other proteins, namely HRG (16.44±3.8 ug/mL vs 9.84±1.74 ug/mL, P = 0.0942), ALB (45.85±2.15 ug/mL vs 48.20±1.68 ug/mL, P = 0.1466) and SCGB1D2 (387.67±36.55 ng/mL vs 482.24±56.04 ng/mL, P = 0.0855) did not change significantly (Figure 3A). Additionally, ORM (P<0.01), FGA (P<0.05) and APOH (P<0.01) that belonged to the *acute phase response pathway* were significantly decreased in the 10 HR but not in the 10 LR before and after GC treatment (Figure 3B).

## Discussion

GCs are among the most effective treatments for allergy and other infammatory diseases [15], [16]. The problems that motivated this study were that some patients show little or no response to GCs, and the need to find markers to monitor that response [4], [17], [18], [19], [20]. In this study, we focused on SAR and aimed to indentify novel biomarkers for GC treatment response. Decades-long research has shown that identification of nasal fluid biomarkers for GC treatment response is complicated by the involvement of many inflammatory cells and mediators in a complex immunological network [15], [16], [21]. On top of this complexity there are considerable individual variations. Studies of asthma and other inflammatory diseases indicate that such variations may be linked to response to GC treatment [2]. Proteomics can be used to profile the protein content of nasal fluids and multivariate analysis to identify combinations of proteins with potential diagnostic value. None of these methods, however, give any information about the functions of the proteins. Pathway analysis helps to obtain a functional overview of the proteins, and thereby to prioritize diagnostic combinations of those proteins [12], [13]. To our knowlegde these methods have not been previously integrated to find diagnostic markers for treatment response. We hypothesized that proteins that correlated to discrimination between HR and LR would be candidate biomarkers for GC treatment. By combining proteomics and multivariate analyses we identified a combination of nasal fluid proteins that did correlate with the discrimination between HR and LR. Further multivariate and pathway analysis of that combination led to the identification of several novel biomarkers for GC treatment response.

We profiled nasal fluid proteins in HR and LR with a LC-MS/MS-based quantitative proteomics analysis, which allows the simultaneous identification and quantification of thousands of proteins in biological samples. Previous proteomic studies have shown that up to 450 proteins are found in nasal fluids from patients with SAR [12], [22]. Here we performed the proteomics analysis on a LTQ Orbitrap Velos instrument, which delivers a high resolution and mass accuracy. This led to the identification of more than 900 nasal fluid proteins. In agreement with our previous study [12], the nasal fluid proteins from patients with SAR were most significantly enriched in the *acute phase response* and *complement signalling* pathways.

Proteomic studies may be performed on pooled samples, in order to find candidate proteins for more detailed analyses of individual samples [3]. On the other hand, multiple nasal fluid proteins might differ in combination to form discrimination patterns between different subgroups of patients. In order to identify such combinations, examination of individual samples are needed. In this study, we speculated that variations in GC treatment response might be due to individul pre-treatment differences in the inflamatory response. We therefore performed the proteomic analysis on individual samples from symptomatic HR and LR. Another issue is that combinations of discriminatory proteins are difficult to infer by univariate analysis. To address this problem, we used OPLS-DA to identify and rank nasal fluid proteins that correlated to the discrimination between HR and LR [12], [13]. Pathway analysis showed that these proteins were most enriched in the *acute phase response pathway*. All the proteins were higher in HR compared to LR, indicating that the *acute phase response pathway* was more active in HR. Pre-treatment differences in the inflammatory response between symptomatic HR and LR may explain the variability in biomarkers for treatment response. This was further confirmed by the ELISA analysis with nasal fluids from HR and LR before and after GC treatment. Three proteins ORM (ORM1/ORM2), APOH and FGA from the *acute phase response pathway* were differentially expressed in HR but not in LR.

We found highly significant decreases of ORM, FGA, APOH, SERPINB3 and CTSD, but not HRG, ALB and SCGB1D2. Among these eight proteins, ALB, A2M and APOH have previously been reported by others and us to decrease following GC treatment in some, but not all studies of SAR [4], [23]. ORM is an acute phase serum protein, which is synthesized by liver as well as epithelial cells and macrophages [24], [25]. In addition, it is found to be a secondary granule protein of neutrophils, which is released immediately in response to activation [26], [27]. This indicates that it exerts immunomodulatory activities not only systemically but also locally during the acute phase immune response. SERPINB3 has been shown to be upregulated in bronchial epithelial cells from asthma patients by Th2 cytokines IL-4 and IL-13 [28].

Analysis of proteins in nasal fluids has the advantage that the findings reflect changes in the affected organ. A problem, however, is that the protein concentrations may vary due because of dilution due to plasma transudation, secretion or the method to collect nasal fluids. On the other hand, differential cell counts or protein ratios (which are unaffected by dilution) and symptom scores show a similar variability, indicating that the variability reflects disease-associated processes. Moreover, since multivariate analysis aims to find altered relations between proteins, rather than absolute changes of individual proteins the possible confounding effects of variable dilution are reduced.

Limitations of this study include that proteomics analysis has limited ability in detecting low-abundance proteins, some of which may have diagnostic potential. Pathway-based analyses can be confounded by limited knowledge about pathways and how those differ between cells and tissues. It is also of note that lack of compliance to treatment may affect the results. As a control, we therefore measured a known biomarker for GC treatment in allergy, namely ECP [7], [29], which was found to decrease significantly. However, further studies of larger materials are needed to assess the clinical value of the candidate biomarkers found in this study. Another possible future research direction is suggested by the pre-treatment differences in proteins between HR and LR. To our knowlegde, such differences have not been previously examined. Although our material was relatively small and HR and LR only partially separated by the proteins, elucidation of such differences could have an important diagnostic implication, namely to predict response to GCs and possibly to other treatments. Ideally, diagnostic protein combinations could be identified in order to routinely determine the optimal medication for individual patients. This would be a step towards personalized treatment in SAR and other allergic diseases.

In conclusion, we identified several novel biomarkers for GC treatment response in SAR with combined proteomics, multivariate and pathway analysis. The analytical principles may be generally applicable to identify biomarkers in clinical studies of complex diseases.

## Materials and Methods

### Ethics statement

The study was approved by the Ethics Committee of the Medical Faculty of the University of Gothenburg. Written informed consents and questionnaire data sheets were obtained from all patients.

### Subjects

40 patients with SAR were included in the study. Their median (range) age was 23 (17–49) and 24 were women. SAR was defined by a positive seasonal history and a positive skin prick test or by a positive ImmunoCap Rapid (Phadia, Uppsala, Sweden) to birch and/or grass pollen. Patients with perennial symptoms or asthma were not included. Nasal lavage samples from the patients were obtained after the start of symptoms during the pollen season, and after 2 weeks of treatment with two doses of 50 µg per dose fluticasone nasal spray in each nostril once daily. All patients were asked to mark their symptoms (rhinorrhea, congestion, and itching) on a visual analogue scale of 10, before and after treatment with fluticasone. These values were added to the total symptom score, as previously described [7].

High-responders (HR) and low-responders (LR) to treatment with GCs were defined as follows. For each patient the ratio between the total symptom before and after GC treatment was computed. HR were defined as the ten patients with the highest ratios, while LR were defined as the ten patients with the lowest ratios.

### Quantitative proteomic analysis of nasal fluids from HR and LR

Nasal fluids from 10 HR and 10 LR during the season before GC treatment were selected for quantitative proteomic analysis. Briefly, all nasal fluids samples were lyophilized, dissolved, alkylated and digested with trypsin. Each five-plex set, using five reporters from a six-plex, consisting of one pooled standard sample and four nasal fluid samples were labelled with Tandem Mass Tag (TMT) reagents respectively following manufacturer's instructions (Pierce, Rockford, IL, USA). Nano LC-MS/MS analysis was performed on a LTQ Orbitrap Velos instrument (Thermo Fisher Scientific, Inc., Waltham, MA, USA) interfaced with an in-house constructed nano-LC column. MS data analysis was performed using Proteome Discoverer version 1.2 (Thermo Fisher Scientific, Inc., Waltham, MA, USA). Details were described in Methods S1.

### Analysis of nasal fluid proteins with ELISA

Proteins were examined by ELISA in nasal fluids from 40 patients with SAR before and after treatment with GCs. Orosomucoid 1/ Orosomucoid 2 (ORM1/ORM2) was analyzed with an ELISA kit from R&D Systems Inc. (Minneapolis, MN, USA). Albumin (ALB) was analyzed with an ELISA kit from Bethyl Laboratories (Montgomery, TX, USA). Apoliprotein H (ApoH) was analyzed with an ELISA kit from United States Biological (Swampscott, MA, USA). Cathepsin D (CTSD), secretoglobin, family 1D, member 2 (SCGB1D2), fibrinogen alpha chain (FGA) and serpin peptidase inhibitor, clade B, member 3 (SERPINB3) were analyzed with ELISA kits from Uscnlife Life Sciences and Technology (Wuhan, China). Histidine-rich glycoprotein (HRG) was analyzed with an ELISA kit from Cusabio Biotech Co., Ltd (Wuhan, China). ECP was analyzed with an ELISA kit from IG Instrumenten-Gesellschaft AG (Zürich, Switzerland). All experiments were performed according to the manufacturers' protocols.

### Pathway, multivariate and statistical analyses

The Ingenuity Pathways Analysis (IPA) software was used to map the proteins onto known canonical pathways [12]. A Fisher's exact test was used to calculate a P value determining the probability that the association between the proteins in the dataset and the canonical pathway is explained by chance alone. Pathways with a P value less than 0.05 were considered to be statistically significant. OPLS-DA was performed in SIMCA-P+ 12.0.1 software (UMETRICS, Umeå, Sweden) to interpret differences in nasal fluid protein profiles between symptomatic HR and LR. Prior to OPLS-DA modelling, proteomics data were pre-processed with log-transformation and unit variance (UV) scaling. OPLS-DA is a supervised multiple regression analysis for classification in which systematic variation in the X block (proteomics data) is divided into two model parts, plus the residual noise: the first part which models the X variation correlated to Y variable and is referred to as the predictive component and the other part which comprise the X variation that is un-correlated to the discriminant Y variable and is referred to as the orthogonal component [10], [30], as judged by a leave out data cross validation (all data are left out once in a 7 leave out series). In the present study the X variation correlated to Y was modelled in the first component, which is referred to as the predictive component. The cross validated, i.e. jack knifed, loadings were used to select candidate proteins that best contributed to the discrimination between responders versus non-responders [30]. As shown in Fig. 2B, 40 proteins were indicated for significant contribution, as indicated by their confidence intervals, and from them the most promising 8 were selected for further analyses and corroboration. The Wilcoxon matched pairs signed ranks test was performed to compare two paired groups. A P value less than 0.05 was considered significant.

## Supporting Information

Methods S1 **Quantitative proteomic analysis of nasal fluids from HR and LR.**
(DOC)

Table S1 **Characteristics of HR and LR.**
(XLS)

Table S2 **The top 40 proteins that correlated to the discrimination between HR and LR.**
(XLS)

## References

[1] Bousquet J, Schunemann HJ, Zuberbier T, Bachert C, Baena-Cagnani CE (2010). Development and implementation of guidelines in allergic rhinitis - an ARIA-GA2LEN paper.. Allergy.

[2] Barnes PJ, Adcock IM (2009). Glucocorticoid resistance in inflammatory diseases.. Lancet.

[3] Schutzer SE, Angel TE, Liu T, Schepmoes AA, Clauss TR (2011). Distinct cerebrospinal fluid proteomes differentiate post-treatment lyme disease from chronic fatigue syndrome.. PLoS One.

[4] Diamant Z, Boot JD, Mantzouranis E, Flohr R, Sterk PJ (2010). Biomarkers in asthma and allergic rhinitis.. Pulm Pharmacol Ther.

[5] Wang WY, Boot JD, Mascelli MA, Gerth van Wijk R, Diamant Z (2009). Comparison of biomarkers between allergic rhinitis only and allergic rhinitis with concomitant asthma.. Allergy.

[6] Benson M, Carlsson L, Adner M, Jernas M, Rudemo M (2004). Gene profiling reveals increased expression of uteroglobin and other anti-inflammatory genes in glucocorticoid-treated nasal polyps.. J Allergy Clin Immunol.

[7] Benson M, Strannegard IL, Strannegard O, Wennergren G (2000). Topical steroid treatment of allergic rhinitis decreases nasal fluid TH2 cytokines, eosinophils, eosinophil cationic protein, and IgE but has no significant effect on IFN-gamma, IL-1beta, TNF-alpha, or neutrophils.. J Allergy Clin Immunol.

[8] Wang H, Barrenas F, Bruhn S, Mobini R, Benson M (2009). Increased IFN-gamma activity in seasonal allergic rhinitis is decreased by corticosteroid treatment.. J Allergy Clin Immunol.

[9] Frese CK, Altelaar AF, Hennrich ML, Nolting D, Zeller M (2011). Improved Peptide Identification by Targeted Fragmentation Using CID, HCD and ETD on an LTQ-Orbitrap Velos.. J Proteome Res.

[10] Whelehan OP, Earll ME, Johansson E, Toft M, Eriksson L (2006). Detection of ovarian cancer using chemometric analysis of proteomic profiles.. Chemometrics and intelligent laboratory systems.

[11] Moazzami AA, Andersson R, Kamal-Eldin A (2010). Changes in the metabolic profile of rat liver after alpha-tocopherol deficiency as revealed by metabolomics analysis.. NMR Biomed.

[12] Wang H, Chavali S, Mobini R, Muraro A, Barbon F (2011). A pathway-based approach to find novel markers of local glucocorticoid treatment in intermittent allergic rhinitis.. Allergy.

[13] de Jong EP, Xie H, Onsongo G, Stone MD, Chen XB (2010). Quantitative proteomics reveals myosin and actin as promising saliva biomarkers for distinguishing pre-malignant and malignant oral lesions.. PLoS One.

[14] Thysell E, Surowiec I, Hornberg E, Crnalic S, Widmark A (2010). Metabolomic characterization of human prostate cancer bone metastases reveals increased levels of cholesterol.. PLoS One.

[15] Bousquet J, Khaltaev N, Cruz AA, Denburg J, Fokkens WJ (2008). Allergic Rhinitis and its Impact on Asthma (ARIA) 2008 update (in collaboration with the World Health Organization, GA(2)LEN and AllerGen).. Allergy.

[16] Okano M (2009). Mechanisms and clinical implications of glucocorticosteroids in the treatment of allergic rhinitis.. Clin Exp Immunol.

[17] Boot JD, Chandoesing P, de Kam ML, Mascelli MA, Das AM (2008). Applicability and reproducibility of biomarkers for the evaluation of anti-inflammatory therapy in allergic rhinitis.. J Investig Allergol Clin Immunol.

[18] Sousa AR, Lane SJ, Cidlowski JA, Staynov DZ, Lee TH (2000). Glucocorticoid resistance in asthma is associated with elevated in vivo expression of the glucocorticoid receptor beta-isoform.. J Allergy Clin Immunol.

[19] Barnes PJ, Greening AP, Crompton GK (1995). Glucocorticoid resistance in asthma.. Am J Respir Crit Care Med.

[20] Kirwan JR (2007). Glucocorticoid resistance in patients with rheumatoid arthritis.. Scand J Rheumatol.

[21] Woodfolk JA (2007). T-cell responses to allergens.. J Allergy Clin Immunol.

[22] Benson LM, Mason CJ, Friedman O, Kita H, Bergen HR (2009). Extensive fractionation and identification of proteins within nasal lavage fluids from allergic rhinitis and asthmatic chronic rhinosinusitis patients.. J Sep Sci.

[23] Meyer P, Andersson M, Persson CG, Greiff L (2003). Steroid-sensitive indices of airway inflammation in children with seasonal allergic rhinitis.. Pediatr Allergy Immunol.

[24] Fournier T, Bouach N, Delafosse C, Crestani B, Aubier M (1999). Inducible expression and regulation of the alpha 1-acid glycoprotein gene by alveolar macrophages: prostaglandin E2 and cyclic AMP act as new positive stimuli.. J Immunol.

[25] Crestani B, Rolland C, Lardeux B, Fournier T, Bernuau D (1998). Inducible expression of the alpha1-acid glycoprotein by rat and human type II alveolar epithelial cells.. J Immunol.

[26] Theilgaard-Monch K, Jacobsen LC, Rasmussen T, Niemann CU, Udby L (2005). Highly glycosylated alpha1-acid glycoprotein is synthesized in myelocytes, stored in secondary granules, and released by activated neutrophils.. J Leukoc Biol.

[27] Gunnarsson P, Levander L, Pahlsson P, Grenegard M (2007). The acute-phase protein alpha 1-acid glycoprotein (AGP) induces rises in cytosolic Ca2+ in neutrophil granulocytes via sialic acid binding immunoglobulin-like lectins (siglecs).. FASEB J.

[28] Yuyama N, Davies DE, Akaiwa M, Matsui K, Hamasaki Y (2002). Analysis of novel disease-related genes in bronchial asthma.. Cytokine.

[29] Norback D, Walinder R, Wieslander G, Smedje G, Erwall C (2000). Indoor air pollutants in schools: nasal patency and biomarkers in nasal lavage.. Allergy.

[30] Vinay P, Allignet E, Pichette C, Watford M, Lemieux G (1980). Changes in renal metabolite profile and ammoniagenesis during acute and chronic metabolic acidosis in dog and rat.. Kidney int.

