# Supplementary material for: Identification of Novel Biomarkers in Seasonal Allergic Rhinitis by Combining Proteomic, Multivariate and Pathway Analysis
Source: PLoS One. 2011 Aug 24;6(8):e23563. doi: 10.1371/journal.pone.0023563 (PMC3160968; doi:10.1371/journal.pone.0023563)
Supplement: Methods S1 — Quantitative proteomic analysis of nasal fluids from HR and LR. (DOC) [file pone.0023563.s001.doc]

**Quantitative proteomic analysis of nasal fluids from HR and LR**

**Sample preparation**

All Nasal fluid samples (2 x 1.5 ml each) from 10 high responders and 10 low responders were lyophilized and reconstructed in 100 µl triethylammonium bicarbonate (TEAB) 100mM / tube. Each sample of 2 tubes was pooled (200 µl / sample) and quantified using Pierce BCA Protein Assay (Thermo Scientific, Basel, Switzerland). 100 µg total protein of each sample was withdrawn and reduced with 200 mM tris-(2-carboxyethyl)phosphine (TCEP), alkylated with 375 mM iodoacetamide (IAA) and precipitated with six volumes of acetone following manufacturer’s instructions (Pierce, Rockford, IL, USA). The pellets were dissolved in 2% SDS and 200 mM TEAB, 5% acetonitrile and digested with trypsin (Promega, Madison, WI, USA) over night in 37 ºC. Each five-plex set consisting of one pooled standard sample and 4 different samples, were then labeled with the TMT® reagent 126, 127, 128, 129, 130 or 131 respectively following manufacturer’s instructions. After quenching of the labeling process, the samples were combined into each set and prepared for fractionation.

**Strong cation exchange chromatography (SCX) of TMT-labeled peptides**

The concentrated peptides were acidified by 10% formic acid and diluted with SCX solvent A (25 mM ammonium formate, pH 2.8, 20% acetonitrile (ACN)) and injected onto a PolySULFOETHYL A SCX column (2.1 mm i.d. × 10 cm length, 5 μm particle size, 300 Å pore size). SCX chromatography and fractionation was carried out on an ÄKTA purifier system (GE Healthcare, Waukesha, WI, USA) at 0.25 mL/min flow rate using the following gradient: 0% B (500 mM ammonium formate, pH 2.8, 20% ACN) for 5 min; 0-40% B for 20 min; 40-100% B for 10 min and 100% B held for 10 min. UV absorbance at 254 nm and 280 nm was monitored while fractions were collected at 0.5 mL intervals and dried down in a SpeedVac. The twelve peptide containing fractions were desalted on PepClean C18 spin columns according to manufacturer’s instructions (Thermo Fisher Scientific, Inc., Waltham, MA, USA).

**LC-MS/MS analysis on LTQ-Orbitrap Velos instrument**

The desalted and dried fractions were reconstituted into 0.1% formic acid and analyzed on a LTQ-Orbitrap Velos (Thermo Fisher Scientific, Inc., Waltham, MA, USA) interfaced with an in-house constructed nano-LC column. Two-micro liter sample injections were made with an Easy-nLC autosampler (Thermo Fisher Scientific, Inc., Waltham, MA, USA), running at 200 nL/min. The peptides were trapped on a precolumn (45 x 0.075 mm i.d.) and separated on a reversed phase column, 200 x 0.075 mm, packed in-house with 3 μm Reprosil-Pur C18-AQ particles. The gradient was as followed; 0-97 min 5-40% acetonitrile (ACN), 0.1% formic acid, 97-102 min 40-90% ACN, 0.1% formic acid and the last 8 min at 90% ACN, 0.1% formic acid.

LTQ-Orbitrap Velos settings were: spray voltage 1.4 kV, 1 microscan for MS1 scans at 60000 resolutions (m/z 400), full MS mass range m/z 400-2000. The LTQ-Orbitrap Velos was operated in a data-dependent mode, that is, one MS1 FTMS scan precursor ions followed by CID (collision induced dissociation) and HCD (high energy collision dissociation), MS2 scans of the five most abundant doubly or triply protonated ions in each FTMS scan. The settings for the MS2 were as follows: 1 microscans for HCD-MS2 at 7500 resolution (at m/z 400), mass range m/z 100-2000 with a collision energy of 50%, 1 microscans for CID-MS2 with a collision energy of 30%.

**Database search and quantification**

MS raw data files from all 12 SCX fractions for one 5-plex TMT set were merged for relative quantification and identification using Proteome Discoverer version 1.2 (Thermo Fisher Scientific, Inc., Waltham, MA, USA). Database search for each set were performed by Mascot search engine using the following critera: Homo sapiens in Swissprot version October 2010, MS peptide tolerance as 5 ppm, MS/MS tolerance as 0.05 Da, trypsin digestion allowing 2 missed cleavages with variable modifications; methionine oxidation, cysteine carbamidomethylation, and fixed modifications; N-terminal TMT6plex, lysine TMT6plex. The detected protein threshold in the software was set to 99% confidence and identified proteins were grouped by sharing the same sequences to minimize redundancy. The median (range) FDR is 0.5% (0.2% - 0.7%) except one set with a FDR = 2.9%.

For TMT quantification, the ratios of the TMT reporter ion intensities in MS/MS spectra (m/z 126-131) from raw data sets were used to calculate fold changes between samples. Ratio were derived by Proteome Discoverer version 1.2 using the following criteria: fragment ion tolerance as 50 ppm for the most confident centroid peak, TMT reagent purity corrections factors are used and missing values are replaced with minimum intensity. Only peptides unique for a given protein were considered for relative quantitation, excluding those common to other isoforms or proteins of the same family. The quantification was normalized using the protein median. The results were then exported into Excel for manual data interpretation.
